# Learning Semantic Graphics Using Convolutional Encoder–Decoder Network for Autonomous Weeding in Paddy

**DOI:** 10.3389/fpls.2019.01404

**Published:** 2019-10-31

**Authors:** Shyam Prasad Adhikari, Heechan Yang, Hyongsuk Kim

**Affiliations:** ^1^Division of Electronics Engineering, Intelligent Robots Research Center (IRRC), Chonbuk National University, Jeonju, South Korea; ^2^Division of Electronics and Information Engineering, Chonbuk National University, Jeonju, South Korea

**Keywords:** semantic graphics, convolutional neural network, autonomous weeding, crop line extraction, encoder–decoder network

## Abstract

Weeds in agricultural farms are aggressive growers which compete for nutrition and other resources with the crop and reduce production. The increasing use of chemicals to control them has inadvertent consequences to the human health and the environment. In this work, a novel neural network training method combining semantic graphics for data annotation and an advanced encoder–decoder network for (a) automatic crop line detection and (b) weed (wild millet) detection in paddy fields is proposed. The detected crop lines act as a guiding line for an autonomous weeding robot for inter-row weeding, whereas the detection of weeds enables autonomous intra-row weeding. The proposed data annotation method, semantic graphics, is intuitive, and the desired targets can be annotated easily with minimal labor. Also, the proposed “extended skip network” is an improved deep convolutional encoder–decoder neural network for efficient learning of semantic graphics. Quantitative evaluations of the proposed method demonstrated an increment of 6.29% and 6.14% in mean intersection over union (mIoU), over the baseline network on the task of paddy line detection and wild millet detection, respectively. The proposed method also leads to a 3.56% increment in mIoU and a significantly higher recall compared to a popular bounding box-based object detection approach on the task of wild–millet detection.

## Introduction

The resurgence of neural networks in the form of “deep” neural networks (DNNs) (Krizhevsky et al., 2012) has dramatically improved the performance of various computer vision tasks such as image classification (Simonyan and Zisserman, 2014; Szegedy et al., 2015; He et al., 2016; Huang et al., 2017), object detection and localization (Ren et al., 2015; Redmon et al., 2016; He et al., 2017), and semantic segmentation (Long et al., 2015; Ronneberger et al., 2015; Badrinarayanan et al., 2017).

Recently, DNNs have also been used extensively for problems in agriculture. Researchers have applied deep learning in agriculture to automate different tasks such as plant recognition (Grinblat et al., 2016), crop type classification (Kussul et al., 2017), plant disease classification (Mohanty et al., 2016; Fuentes et al., 2018), weed identification (Dyrmann et al., 2016; Dyrmann et al., 2017), and land cover classification (Kussul et al., 2017; Ienco et al., 2017). Agricultural farm is a semi-constrained environment which is easier than unconstrained natural environments for the adoption of DNN. However, application of DNN to agriculture has its own challenges because of confusion due to low variation between the target classes. Crops and weeds are similar in shape, texture, color, and position, which results in significant reduction in accuracy of DNN systems (Mohanty et al., 2016; Dyrmann et al., 2016). Furthermore, severe overlapping and occlusion, a common phenomenon in the farm, also poses serious challenges to the application of DNN in agriculture. Among the different areas for the use of DNN in agriculture, plant and weed identification has received much attention in the literature due to its enormous practical impact. This study is focused on the use of DNN in rice fields.

Rice is a widely eaten staple food by billions of people around the world. It is considered the lifeline of the Asia-Pacific region where 90% of the world’s rice is consumed. With increasing population, the demand for rice is expected to grow, and the challenge is to increase the production of rice using limited land, water, and manpower and less use of agrochemicals. One of the factors responsible for reduced rice yield is weeds. Weeds are aggressive growers which compete for nutrition and other resources and thus reduce production. Moreover, weeds serve as hosts to pests and diseases that are otherwise harmful for the crop. Various weed control methods like hand weeding, mechanical weeding, chemical weeding, and biological control are available for weed management. Herbicides are used extensively to manage weeds; however, their increasing use has inadvertent consequences to the human health and the environment. Though mechanical weeding saves farmers from the drudgery of hand weeding, it is nonetheless labor-intensive. With a decline in interest among the younger generation to join agriculture, the available manpower for labor is limited. Biological control methods using fish, insects, and birds are environmentally friendly and used for effective weed management in organic rice cultivation.

With the advancements in robotics, autonomous agricultural robots have been widely adopted to increase crop productivity and improve labor efficiency. Machine vision-based systems have been used in autonomous agricultural robots for weed management in row crops like rice and maize (Guerrero et al., 2017; Ma et al., 2019). Navigation systems are a crucial part of such autonomous robots where a guidance line has to be computed to guide the robot for weed control. Vision sensor-based autonomous guidance systems have been widely researched for extracting the crop lines to guide the robot (Choi et al., 2015; Zhang et al., 2017).

In this work, we used data from a row-transplanted organic rice field in the Republic of Korea where the golden apple snail (*Pomacea canaliculata*) was used for biological control of weeds. The golden apple snail is effective in controlling most of the weeds except for the wild millet. Wild millet being similar in appearance to the rice plant makes it difficult for hand weeding. Towards the end goal of an autonomous weeding system for paddy, we present a DNN-based system to (a) automatically detect rows of crop and (b) detect weed (willet millet) in row-sown (transplanted) paddy field. The detected crop lines act as a guiding line for an autonomous weeding robot for inter-row weeding, whereas the detection of weeds enables autonomous intra-row weeding.

## Related Work

### Crop Line Detection

Previous works on detecting crop rows using vision-based systems primarily detect the position of the crops using different handcrafted features like living tissue indicators (Søgaard and Olsen, 2003), vegetation index (Bakker et al., 2008; Montalvo et al., 2012), morphological features (Choi et al., 2015), and extraction of the crop line using different pattern recognition and machine learning techniques like distribution of pixel values, vanishing point detection, Hough transform, and linear regression (Søgaard and Olsen, 2003; Bakker et al., 2008; Montalvo et al., 2012; Choi et al., 2015; Jiang et al., 2016).

Methods based on handcrafted features work well under controlled conditions; however, they can fail to work in real farm conditions, as it is practically infeasible to hand-engineer features which capture the extensive diversity found in real farm environments. The methods based on color index work well in the absence of weeds in between the rows, as the vegetation index or living tissue index of weeds is similar to that of crops. The presence of weeds and different natural conditions like shades or light reflection affects the extraction of binary morphological features, which ultimately affects the accuracy of the extracted crop line.

Recent advancements in neural networks have demonstrated that automatic feature learning using convolutional neural networks (CNNs) are more successful than hand-engineered features. Methods based on CNNs have produced state-of-the-art results in different computer vision and pattern recognition problems like object detection and classification (Ren et al., 2015; Redmon et al., 2016; Huang et al., 2017) and semantic segmentation (He et al., 2016).

**Figure 1:**
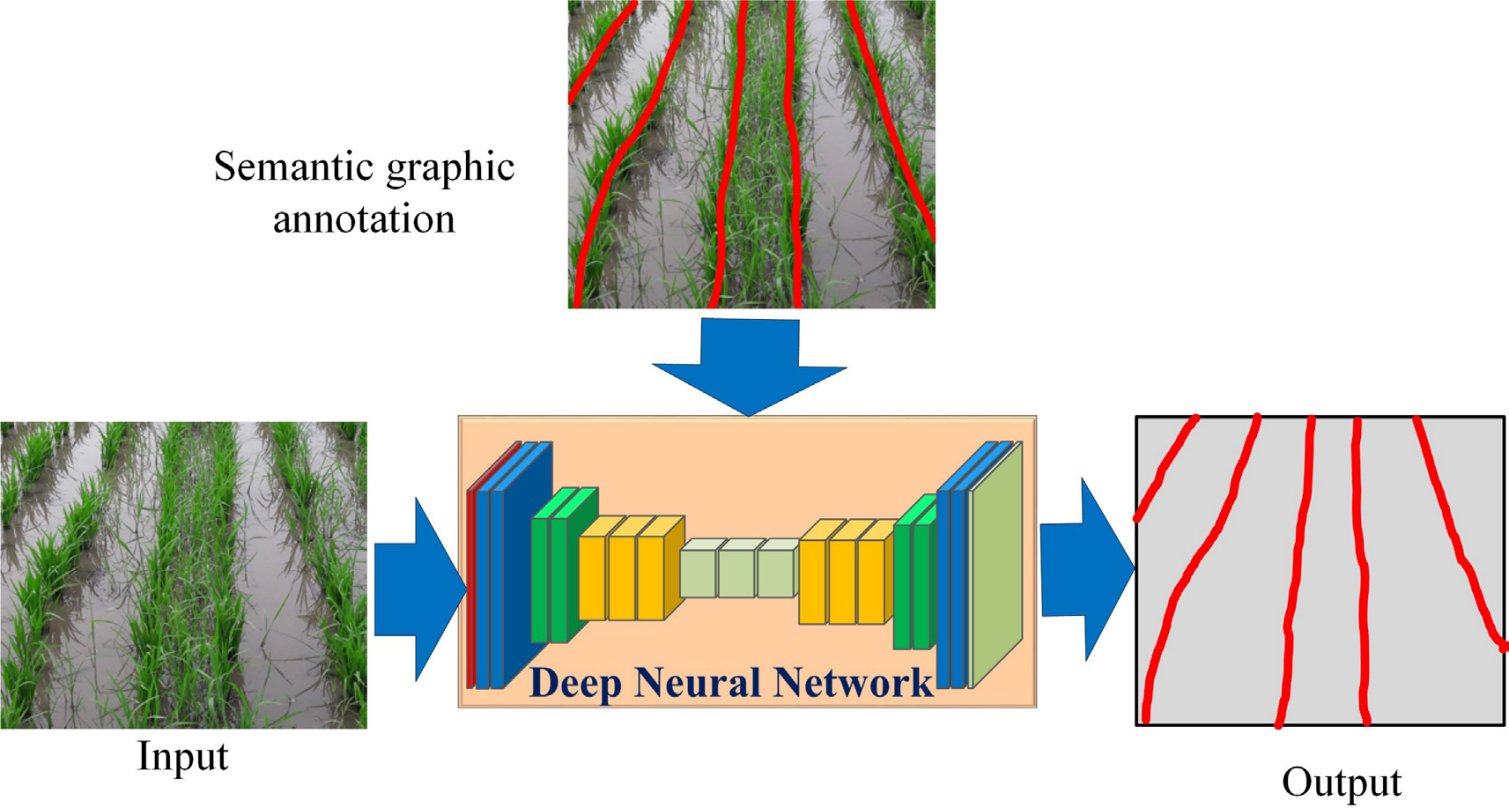
The proposed approach of training deep neural networks to learn the concept of crop line using semantic graphics.

In this work, we use CNN to extract the crop lines. Unlike prior works which segment the input into different regions and extract the crop lines, we propose to train a CNN to directly learn the concept of a crop line using “semantic graphics” as shown in **Figure 1**.

### Weed Detection

Recently, DNN-based algorithms for classification of weeds and crops have attracted much attention. Two different CNNs were used to segment and classify image pixels into crop and weeds (Potena et al., 2016). A method based on *K*-means feature learning combined with CNN was used for weed identification in soybean seedlings (Tang et al., 2017). A fully CNN was used to detect single weed instances in image from winter wheat fields with leaf occlusion (Dyrmann et al., 2017). CNN-based semantic segmentation approaches to separate crops, weeds, and background have also been studied (Milioto et al., 2018; Ma et al., 2019). While semantic segmentation-based approaches are helpful for widely spaced crops and weeds, these approaches are difficult to adopt in fields with heavy overlap and occlusion owing to the difficulty in obtaining per-pixel ground truth annotations. Moreover, the difficulty in obtaining ground truth labels is compounded for crop and weeds, like rice and wild millet, which have similar appearances.

In this work, we propose to learn “semantic graphics” using CNN for the identification of rice and wild millet.

### Semantic Graphics

One of the factors enabling the increase in performance of DNNs is the availability of a huge amount of data for training. However, for supervised training of DNNs, the data has to be annotated manually with ground truth. It is expensive and time-consuming to prepare large-scale ground truth annotations (Bearman et al., 2016), and hence, there is a bottleneck in extending the application of DNN to new applications which require the network to be trained on custom datasets. Manual annotation is particularly time-consuming for semantic segmentation where per-pixel annotation is required. Per-pixel semantic labeling is also economically not viable without employing methods which reduce human labor.

To reduce the dependency on large-scale detailed annotations, weakly or semi-supervised learning techniques have been explored in the literature. In the weakly supervised setting, the training images are annotated only at the image level or sparsely annotated at the pixel level, thus requiring less time and effort for annotation. Different forms of weak supervision have also been explored in the literature such as image-level labels (Pinheiro and Collobert, 2015), bounding boxes (Papandreou et al., 2015), and point annotations and free-form scribbles (Bearman et al., 2016; Lin et al., 2016). However, much of the focus in the literature has been towards detecting or segmenting “objects” with a well-defined shape, appearance, and boundary. Less attention has been paid towards understanding complex scenes that are difficult even to annotate correctly due to similar appearance and ambiguous boundaries.

**Figure 2:**
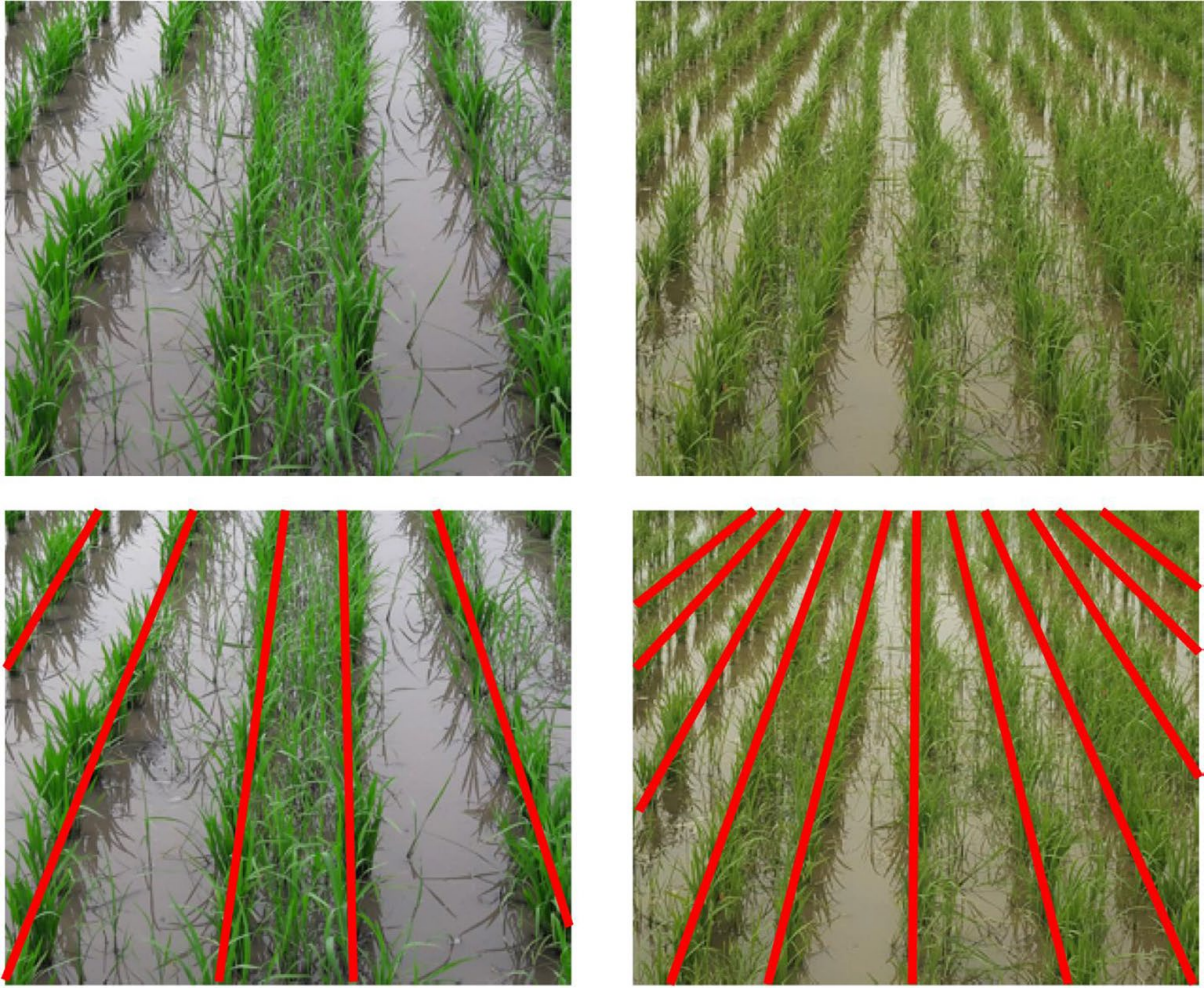
Semantic graphics: (top) images of row-transplanted paddy field. (bottom) Manually marked semantic graphics representing the rows of paddy is superimposed on the original images. Even at places where the paddy lines are rendered indistinguishable due to the heavy presence of weeds, humans can easily figure out the actual lines and represent those using semantic graphics. (Best viewed in color).

To simplify the process of annotating such complex scenes, we introduce the notion of semantic graphics. Semantic graphics is a graphical sketch where a target concept is expressed in the form of a figure for easy learning by neural networks. Semantic graphics can encode human knowledge directly in intuitive graphics which can be annotated with considerable ease even for complex scenes. For example, in the image of a line-transplanted paddy field shown in **Figure 2**, the lines of paddy have been rendered indistinguishable due to high weed pressure. However, humans can easily figure out the actual rows of paddy in the image, including in those regions where the actual demarcation does not exist due to weeds. One of the meaningful ways to mark the rows is by sketching a line as shown at the bottom of **Figure 2**.

Semantic graphics is different from semantic segmentation as pixels belonging to the same semantic region or super-pixel may not be necessarily labeled with the same target category. Semantic graphics is particularly useful for tasks which are otherwise challenging for existing pixel-based semantic segmentation methods. For example, the rows of paddy and the wild millet in between the rows, as shown in **Figure 2**, are semantically similar; therefore, it is difficult and time-consuming to prepare dense per-pixel annotation to be used for semantic segmentation. However, it is easier to figure out the actual crop rows and represent those using semantic graphics. In this work, we demonstrate that semantic graphics are an effective way towards training CNNs to learn higher-order concepts like the crop line and to differentiate between crops and weeds.

### Convolutional Encoder–Decoder Network

A convolutional encoder–decoder network is a standard network used for tasks requiring dense pixel-wise predictions like semantic segmentation (Badrinarayanan et al., 2017), computing optical flow and disparity maps (Mayer et al., 2016), and contour detection (Yang et al., 2016). The encoder in the network computes progressively higher-level abstract features as the receptive fields in the encoder increase with the depth of the encoder. The spatial resolution of the feature maps is reduced progressively *via* a down-sampling operation, whereas the decoder computes feature maps of progressively increasing resolution *via* un-pooling (Zeiler and Fergus, 2014) or up-sampling. The network has the ability not only to model features like shape or appearance of different classes but also to model long-range spatial relationships. This attribute of modeling local and global features makes this architecture suitable for learning semantics graphics, as shown in **Figure 1**.

Different variations of the encoder–decoder network have been explored in the literature for improved performance. Skip connections (Ronneberger et al., 2015) have been used to recover the fine spatial details during reconstruction which get lost due to successive down-sampling operations involved in the encoder. Addition of larger context information using image-level features (Liu et al., 2015), recurrent connections (Pinheiro and Collobert, 2014; Zheng et al., 2015), and larger convolutional kernels (Peng et al., 2017) has also significantly improved the accuracy of semantic segmentation. Other methods studied for improving semantic segmentation accuracy include hierarchical supervision (Chen et al., 2016) and iterative concatenation of feature maps (Jégou et al., 2017).

In this work, we design an enhanced encoder–decoder network, named “extended skip network” (*ESNet*), to learn the semantic graphics. We demonstrate that the enhanced network exhibits significant performance improvement over the baseline network on the problem of crop line detection and weed detection. We also demonstrate that the proposed method has improved performance on the task of weed detection over a popular bounding box-based object detection method.

## Materials and Methods

### Dataset

#### Paddy Line Dataset

The focus of this dataset is to extract the rows of paddy, as shown in **Figure 2**. The detected crop lines will enable the navigation of an autonomous agent in the field to accomplish different agricultural tasks like mechanical weeding and precision spraying of herbicides, pesticides, nutrients, etc. *Paddy line dataset* was prepared to evaluate the proposed method. This dataset consists of 350 images of line-transplanted paddy field captured with a handheld camera while walking between the rows of the crop. The dataset contains different scenarios like unevenly spaced rows, weed-infested fields rendering crop rows indistinguishable, and missing crops in a row which make the problem of detecting rows challenging. The images were captured in three different fields at different geographical locations but during the same phonological stage; tillering. Out of the total 350 images, 300 images were used for training and 50 images were set aside for the test. Due to perspective, the rows of rice appear to converge at the horizon and are indistinguishable. In this study we consider only the near-field view for ease of annotation. The rows of rice were annotated with few-pixel-thick lines as shown in **Figure 2**.

The images were down-sampled to a uniform size of 600 × 600 pixels to reduce computation time and memory requirement. Though this dataset has less number of training images, extensive data augmentation was carried out during training by scaling the original image by a factor sampled randomly in the range [0.5, 1.5], rotating the image by an angle sampled randomly from [−15, 15] degrees, mirroring the image randomly along the vertical axis, randomly distorting the image brightness and saturation, and generating random crops of size 512 × 512.

#### Paddy–Millet Dataset

Paddy and wild millet are similar in appearance; therefore, they are difficult to discriminate. Wild millet are aggressive growers which compete for resources and therefore have to be weeded out for better yield of paddy. The goal is to identify and localize the “weed” wild millet present among the paddy so that an autonomous agricultural robot can eliminate the “weed” while keeping the crop intact.

**Figure 3:**
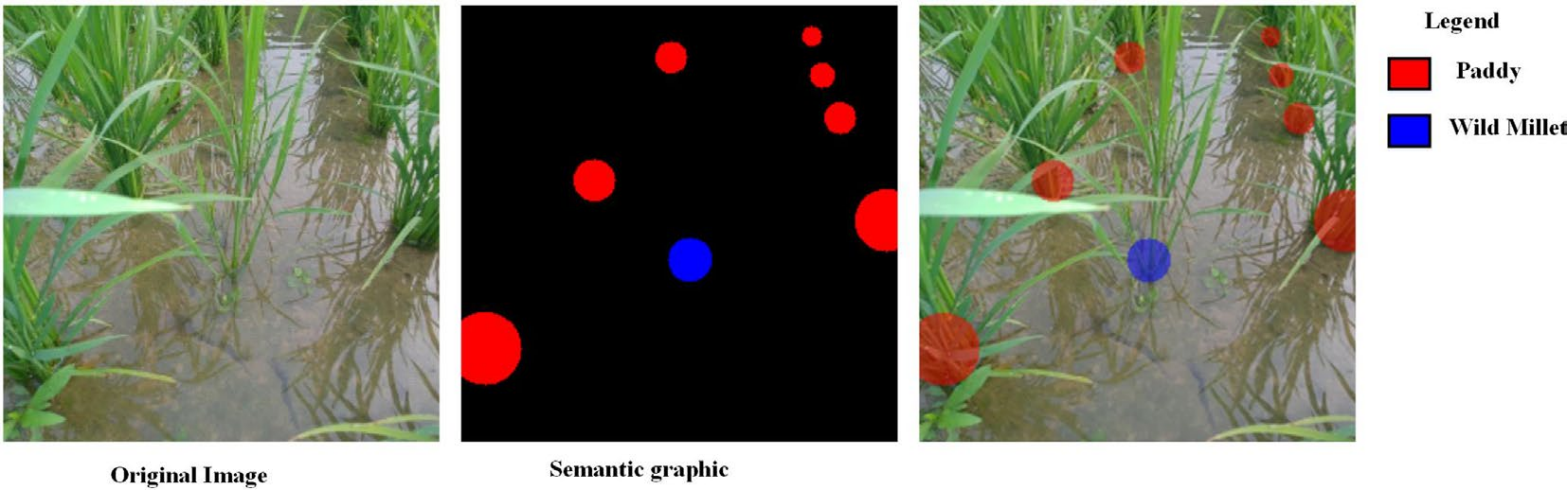
Semantic graphics for paddy–millet dataset. **(A)** Image of a paddy field with wild millet. **(B)** Semantic graphics annotation. The color-filled circles used to annotate the base of the plants indicate multiple meanings such as category of the plant, location of the key-point, and their distance from a viewpoint. **(C)** Annotation superimposed on the source image. (Best viewed in color)

A dataset, namely, *paddy–millet dataset*, consisting of 760 images of row-transplanted paddy field captured with a handheld camera while walking between rows of the crop, as shown in **Figure 3**, was prepared for the experiments. Out of the total 760 images, 660 images were used for training and 100 images were set aside for testing. Semantic graphics was used to annotate the ground truth data and the base of the respective plant categories; namely, paddy and wild millet were the target key-points to be detected. These key-points were annotated with solid circles, and all unmarked pixels were considered as background. The key-points near the camera viewpoint were annotated with bigger radius circles which could extend well beyond the boundary of the key-point whereas the key-points farther away from the viewpoint were annotated with progressively smaller circles. The semantic graphics used to annotate this dataset can represent multiple higher-level meanings such as category of the plant, location of the key-point, and their distance from a viewpoint. However, only the plant category and location of the key-point are considered in this work.

Each high-resolution image was down-sampled to a uniform size of 288 × 288 pixels to reduce computation time and memory requirement. The data were augmented by mirroring the images randomly along the vertical axis and generating random crops of size 256 × 256 during training.

### Architecture of Extended Skip Network

**Figure 4:**
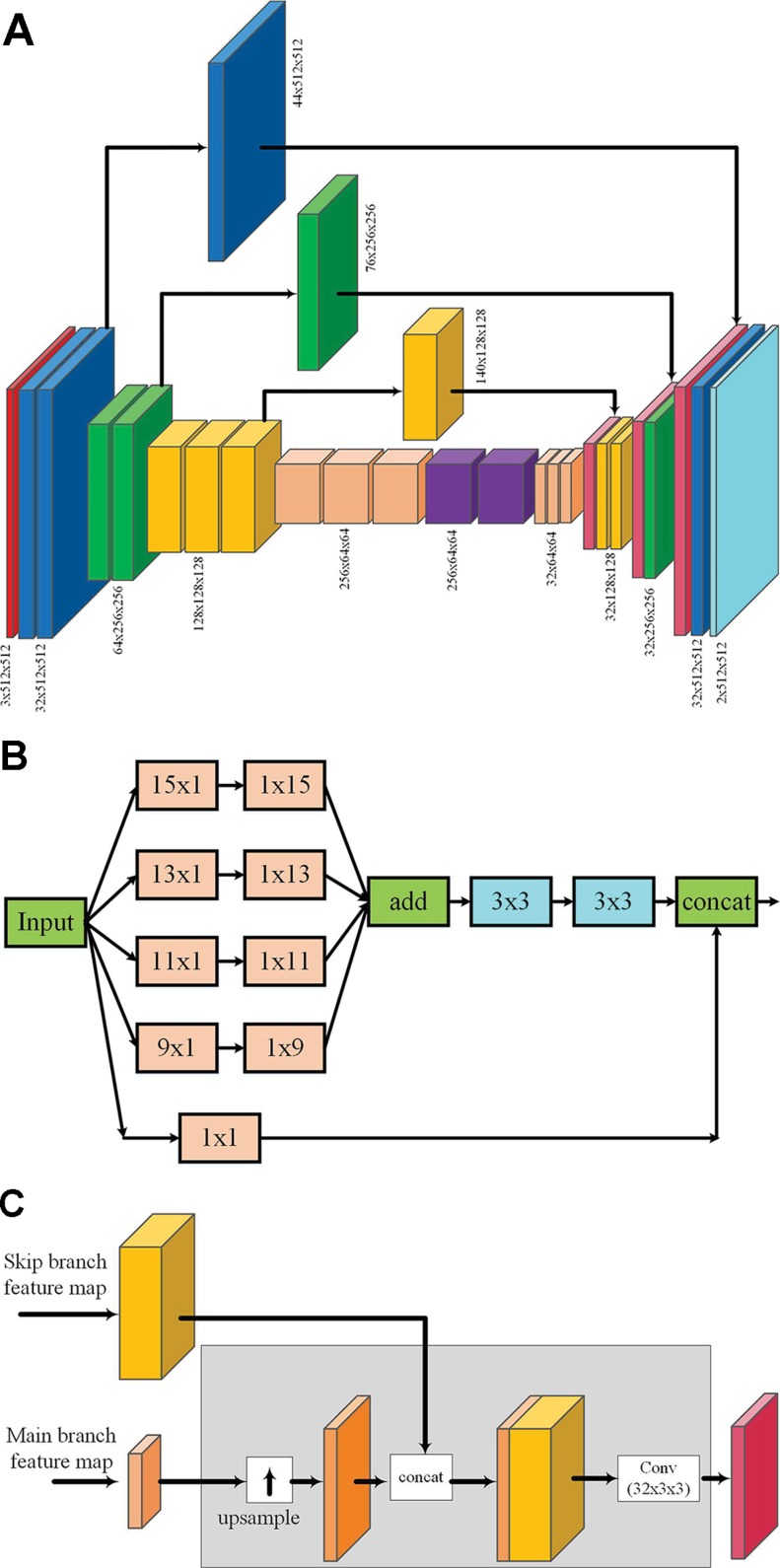
*ESNet*: The proposed extended skip network for end-to-end learning of semantic graphics **(A)**. Diagram representing the output feature maps at each stage of the network **(B)**. Extended skip module: If *Cin* is the number of channels in the input, the output after 1 × 1 convolution has the same number of feature maps as the input, whereas to keep the computational complexity minimal, the number of feature maps at the output after other multi-scale filter banks is kept constant at 12. Hence, the total number of output feature maps of the skip module is (*Cin* + 12). **(C)** The combined operations (up-sampling, concatenation, and convolution with fixed number of output feature maps) involved while merging feature maps from the extended skip module with the decoder. For simplicity, this detailed structure is not shown in **(A)**.

An enhanced fully convolutional encoder–decoder network, called “enhanced skip network” (*ESNet*), as shown in **Figure 4A**, is proposed for end-to-end learning of semantic graphics. The network consists of a contracting encoder and an expanding decoder. The detailed network architecture is given as **Supplementary Material**. The encoder consists of multiple VGGNet-like (Simonyan and Zisserman, 2014) blocks, where each block consists of multiple 3 × 3 convolution followed by batch normalization (Ioffe and Szegedy, 2015) and a nonlinear activation. Each VGG-style block in the encoder, except the last block, is followed by max pooling to reduce the spatial resolution of the feature maps. These blocks are followed by two convolution blocks (with large kernels) → batch normalization → nonlinear activation blocks, which are used at the tail of the encoder to capture a wider context. To reduce the computation overhead, these large convolutions are computed using separable kernels (Jin et al., 2014). The rectified linear unit (ReLU)is used as the nonlinear activation throughout the network.

The decoder is similar in architecture to the encoder but with fewer feature maps for optimized computation and memory requirements. Each block in the decoder is also a repeating structure of up-sampling, followed by multiple 3 × 3 deconvolution, batch normalization, and nonlinear activation operations. The number of feature maps at each level in the decoder is kept constant except for the output layer where it is equal to the number of target classes. The network contains extended skip connections where the feature maps from the encoder are concatenated to the corresponding feature maps in the decoder. The extended skip module consists of a bank of multi-scale filters as shown in **Figure 4B**. The output feature maps of the extended skip module are merged with the corresponding feature maps of the decoder as shown in **Figure 4C**.

The proposed *ESNet* is inspired from and exploits the elements of three different DNNs in a single network, namely, (a) skip layers to recover fine spatial details (Ronneberger et al., 2015), (b) larger convolutional kernels to incorporate a wider image context (Peng et al., 2017), and (c) multi-scale filter bank or “inception” module (Szegedy et al., 2015). However, unlike Ronneberger et al. (2015), whose study used skip layers that are fixed identity connections (copy and concatenate), we propose to make the architecture more general by learning these connections using multi-scale convolution. Large convolutional kernels (Peng et al., 2017) are used to increase the effective receptive field of the network for learning semantic graphics. However, the large kernels are used only at the tail of the encoder and the skip layers.

Finally, the large convolutional kernels in the skip layers are arranged in a multi-scale filter bank module (Szegedy et al., 2015),as shown in **Figure 4B**, to incorporate the required input context during learning without having to empirically find an appropriate kernel size. This module provides multi-scale features which are more efficient for learning semantic graphics than selecting a single-scale context, as will be shown in the results presented in Section Ablation Experiments.

### Training Parameters and Evaluation Metrics

The proposed approach is evaluated on the two problems, paddy line detection and wild millet detection, by training the models from scratch. The DNN models for both the tasks are trained by minimizing the pixel-wise cross-entropy loss given as

(1) $$CELoss=-\frac{1}{\text{N}}\sum_{i=1}^{N}\sum_{c=1}^{L}1_{y_{i}\in L_{\text{c}}}\log\text{p}[{\text{y}}_{i}\in {\text{L}}_{c}]$$

where *N* is the total number of pixels, *L* is the number of semantic categories, $1_{{\text{y}}_{i}\in {\text{L}}_{\text{c}}}$ is a binary indicator function if category *c* is the ground truth label for the *i*th observation, and $\text{p}[{\text{y}}_{i}\in {\text{L}}_{\text{c}}]$ is the predicted probability of the model for that category.

The network shown in **Figure 4** was used for learning the semantic lines. The details of the network are included as **Supplementary Material**. The network was initialized using Xavier initialization (Glorot and Bengio, 2010) and trained on mini-batches of five using the Adam method (Kingma and Ba, 2014) with an exponential decaying learning rate of 10^−4^ for a total of 100 epochs, with all the training images being processed per epoch. As the paddy lines and background pixels are highly imbalanced in each mini-batch, the loss for the two categories is weighted by the class proportion of pixels computed on the training set. The paddy–millet dataset was trained on mini-batches of size 10, with a learning rate of 10^−4^ and a decay factor of 0.94 after successive 10,000 iterations. The network was trained for a total of 60,000 iterations.

The performance of the trained model for both the datasets are evaluated using an intersection-over-union (IoU) metric,

(2) $$IoU=\frac{T\cap P}{T\cup P}$$

where *T* is the target and *P* is the predicted category. In addition to the IoU metric, the precision and recall values for wild millet detection and the average pixel deviation of the predicted line from the ground truth for paddy line detection are also reported. The experiments were conducted in TensorFlow (Abadi et al., 2016) using an NVIDIA Titan-X graphics processing unit (GPU).

### Comparison Models

The proposed *ESNet* is compared to other commonly used CNN architectures which produce image-like outputs like the *UNet* (Ronneberger et al., 2015), *FCN8* (Long et al., 2015), and *DeepLabV3* (Chen et al., 2017). The problem of paddy and wild millet detection can be addressed as a bounding box-based object detection and localization approach of *Faster-RCNN* (Ren et al., 2015) also. Therefore, the proposed network is compared with *Faster-RCNN* on the task of paddy and wild millet detection. We also implement a basic encoder–decoder network (*EDNet*) with a comparatively large number of parameters for comparison on the paddy–millet dataset. The details of the networks used in this study are included as **Supplementary Material**.

In the *Faster-RCNN* setting, the paddy–millet dataset was annotated by replacing the semantic graphics with minimum bounding boxes and the problem was solved as a detection and localization problem. The IoU was then computed on the predicted bounding boxes [proposals with class scores *p* > 0.8 with a non-maximum suppression (NMS) threshold of 0.2] and the ground truth annotation. For a fair comparison with the semantic graphics method, the IoU was computed after substituting each bounding box with a maximal circle that fit the box. The detection accuracy was also evaluated using precision and recall values. Any prediction whose center lay within a distance of *d_thresh* (= 15) pixels from the center of its corresponding ground truth was deemed correct (true positive). The *VGG16* (Simonyan and Zisserman, 2014) model pre-trained on ImageNet (Deng et al., 2009) was used to initialize the *Faster-RCNN* and *EDNet* and fine-tuned on the paddy–millet dataset.

### Post-Processing: Dominant Semantic Line Extraction

The proposed method of detecting crop lines outputs semantic lines for every visible row of paddy. However, for practical purposes, it is often sufficient and meaningful to detect only a few dominant rows, for example, the host rows and a few of its neighbors. Therefore, a simplified random sample consensus (RANSAC) (Fischler and Bolles, 1981) like post-processing step is employed to extract only the four dominant rows. The output semantic graphics is binarized, and the line segments are sorted according to their length. The longest line segment is chosen as a seed, and a straight line is fit to this segment. All the points within a distance of *d_thresh* (= 15) pixels are assigned as inliers to the initial line, and a new estimate of the line is computed. The resultant line after the second iteration is the first dominant line.

After the first line is detected, all the pixels that are inliers to this line and any other line segments with more than 50% pixel inliers to this line are excluded from processing the remaining dominant line segments. The next longest line segment in the binarized output is then chosen as the second seed, and the above procedure is repeated until the required number of dominant lines are extracted.

The accuracy of the extracted dominant lines is computed using mean pixel deviation (*mpd*) from the ground truth line. The *mpd* is computed as the average of the row-wise difference between the predicted line and its corresponding ground truth. Let (*x*_p_, *y*) be a point on the predicted line and (*x*_g_, *y*) be its corresponding point on the ground truth line; then the row-wise pixel deviation (*pd*) is given as *pd* = |*x*_p_ − *x*_g_|. Then *mpd* is the mean of *pd* computed across all the lines in the test set.

## Results and Discussion

### Crop Line Detection

The quantitative results of the proposed network, *ESNet*, along with the results of *UNet*, *FCN8*, and *DeepLabV3* on the paddy line test set are presented in **Table 1**. From **Table 1**, we see that the proposed network achieves the highest mean intersection over union (mIoU, 62.73%) among all the models considered in this study. The mIoU of the proposed method is 6.29%, 4.56%, and 2.38% higher than that of *UNet*, *DeepLabV3*, and *FCN8*, respectively.

**Table 1:** Comparison of different networks on the paddy line dataset.

Method	#parameters (million)	mIoU (%)	fps (512 × 512 pixels, Titan-X GPU)
**UNet**	**∼2.14**	56.44	21.28
**FCN8**	∼38.16	60.35	21.60
**DeepLabV3**	∼4.14	58.17	**31.30**
***ESNet*** **(proposed)**	∼5.74	**62.73**	10.97

ESNet, enhanced skip network; GPU, graphics processing unit; mIoU, mean intersection over union. *The performance is quantified using mIoU*. For Methods, bold is used to highlight the proposed method, whereas bold numbers are used to highlight the best results.

However, the mIoU of the detected semantic lines is less than the mIoU reported on the task of semantic segmentation using similar networks. This is because, unlike the per-pixel ground truth labels used in semantic segmentation, the annotations used for semantic lines are abstract and can be subjective; *i.e.*, annotation of the same line of crop by two human annotators can differ significantly with little overlap between the two. This subjective nature of annotation affects network training and test accuracy. The quantitative analysis on the effect of the subjective nature of annotating semantic graphics is a subject of our future research.

From **Table 1**, we see that the proposed method is slow during inference. Even on a Titan-X GPU, the method runs at 10 fps. This is due to the large-sized kernels used in the network. The bulky Titan-X GPU may not be an optimal choice for use in field robots, and lightweight and more power-efficient GPUs like the Jetson TX2 are more practical. We can expect a considerable slowdown in inference time using the Jetson TX2. However, for a carefully designed system, we can limit the field of view of the vision sensor and restrict the region of interest (ROI) to gain inference speed. From our experiments, it was observed that the proposed network can process 5 fps for an input ROI of 192 × 256. This inference time is expected to be sufficient for any practical application of a slow-moving robot like a tractor running in a flooded rice field.

**Figure 5:**
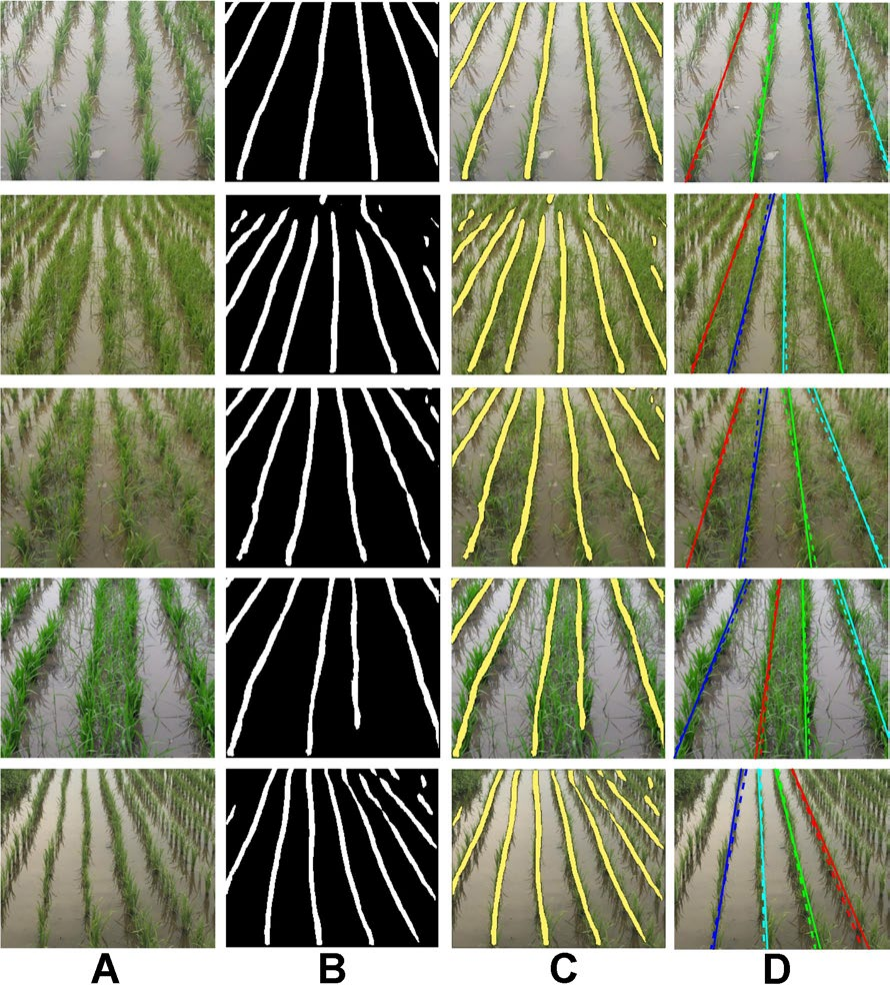
Qualitative results of learning semantic lines using the proposed extended skip network on the paddy line dataset. **(A)** Test images, **(B)** corresponding outputs of the proposed network, **(C)** output superimposed on test image, and **(D)** extracted dominant paddy lines along with the ground truth lines (dotted). (Best viewed in color).

Some qualitative results on the paddy line test set are presented in **Figure 5**. While the proposed method is able to successfully detect paddy line in well-separated crop rows (first and fifth rows), the crop rows are delineated in high-weed-pressure areas also (second, third, and fourth rows). We also see that the line detection accuracy is higher for rows near the principal axis of the camera lens, whereas it is low for rows lying further away. Training the network on a larger dataset is expected to increase the accuracy of the detected lines throughout the image.

However, as explained in Section Post processing: Dominant Semantic Lines Extraction , for the practical purpose of navigating the field, it is often not necessary to detect crop rows lying further away from the principal axis. Some qualitative results of the extracted dominant lines are presented in **Figure 5D**. The detected dominant lines are in close agreement with the ground truth line, which is also evident from the *mpd* values presented in **Table 2**. Though the difference in mIoU of *UNet* and *ESNet* is high, no significant difference in *mpd* is observed between these two networks. The random sample consensus (RANSAC)-based post-processing compensates for the low mIoU of *UNet*.

**Table 2:** Comparison of different networks on the paddy line dataset.

Method	Mean pixel deviation	Deviation [−max, max]
*Unet*	3.39	[−27, 48]
**ESNet** (proposed)	**2.89**	**[−24, 24]**

ESNet, enhanced skip network. *The performance is quantified using mean pixel deviation of the predicted line from the ground truth line. *For Methods, bold is used to highlight the proposed method, whereas bold numbers are used to highlight the best results.

### Wild Millet Detection

The quantitative results of the proposed method on the paddy–millet dataset along with results of *Faster-RCNN*, *EDNet*, *UNet*, *FCN8*, and *DeepLabV3* are presented in **Table 3**. From our experiments, it was observed that initializing *Faster-RCNN* and *EDNet* with *VGG16* weights pre-trained on ImageNet and fine-tuning only the last few layers resulted in low-accuracy networks. However, a significant increase in mIoU was observed when all the layers were fine-tuned. The lower accuracy of the networks with few layers fine-tuned is due to the difference in the type of classes used in the pre-trained *VGG16* model. The generic “object” features extracted by the pre-trained *VGG16* are not optimal to discriminate between the categories used for this dataset.

**Table 3:** Comparison of different variants of *Faster-RCNN* and the proposed method on the paddy–millet dataset.

Method	#parameters (million)	Paddy	Millet	mIoU (%)	Precision (%) (*d_thresh* = 15)		Recall (%) (d_thresh = 15)		F1 score	
					Paddy	Millet	Paddy	Millet	Paddy	Millet
*Faster-RCNN*	∼136	50.07	46.37	48.22	**95.42**	94.69	74.87	68.58	83.90	79.54
*EDNet*	∼15.27	**57.15**	45.52	51.34	90.0	86.29	**92.30**	68.59	**92.19**	76.42
*UNet*	**∼2.14**	48.65	42.62	45.64	91.86	84.37	81.02	69.23	86.10	76.05
*FCN8*	∼38.16	53.30	45.40	49.36	89.29	77.07	89.74	77.56	89.51	77.31
*DeepLabV3*	∼4.14	15.93	43.27	29.61	51.58	**95.69**	33.33	57.05	40.49	71.48
ESNet (proposed)	∼5.74	56.53	**47.02**	**51.78**	87.80	84.56	**92.30**	**80.76**	89.99	**82.16**

EDNet, encoder–decoder network; ESNet, enhanced skip network; mIoU, mean intersection over union. *The performance is quantified using intersection over union (IoU), precision, and recall*. For Methods, bold is used to highlight the proposed method, whereas bold numbers are used to highlight the best results.

Though *EDNet* has a fraction of the parameters, it exhibits an mIoU higher than that of *Faster-RCNN*. This shows that the proposed method can be used to solve the problem of discriminating paddy and wild millet with higher accuracy, fewer parameters, and a simple end-to-end training compared to the existing bounding box approach of object detection. From **Table 3**, we see that the proposed *ESNet* leads to a 0.44% increment in mIoU with significantly less number of parameters than did *EDNet*. We also see that the mIoU of *ESNet* is 22.17%, 6.14%, and 2.42% higher than that of *DeepLabV3*, *UNet*, and *FCN8*, respectively.

Though *Faster-RCNN* has the highest precision, it has poor recall values. On the other hand, *ESNet* has balanced precision and recall values. From an application perspective, though *Faster-RCNN* is less likely to mistake a rice plant as millet, it is more likely to leave a significant number of weeds in the field undetected. However, *ESNet* detects most of the millets present in the field and is also less likely to mistake rice for millet.

**Figure 6:**
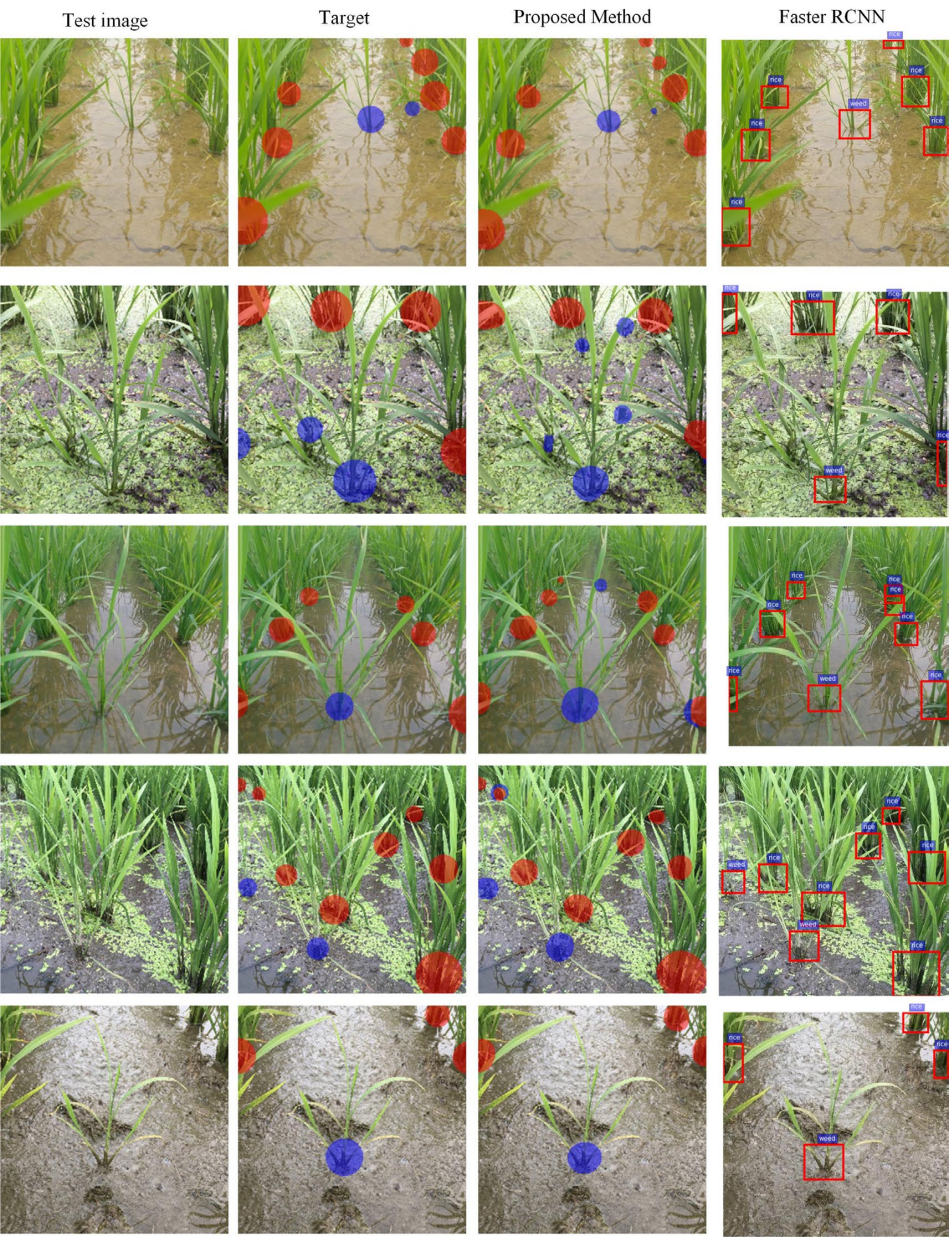
Qualitative results of learning semantic graphics using the proposed convolutional encoder-decoder network on paddy–millet dataset. (Best viewed in color).

Some qualitative results on the paddy–millet dataset are presented in **Figure 6**. While *ESNet* detects most of the millets in the field, it also produces some false positives (second and third rows). Some failure cases (third and fourth rows) are also observed where there is overlap between the two classes. Training the network with a larger dataset is expected to increase the accuracy of the system and reduce the number of failure cases. Though no post-processing has been implemented in the current study, these failure cases can also be reduced by using morphology-based post-processing operations like erosion and filtering.

## Ablation Experiments

The effectiveness of the proposed *ESNet* is evaluated by comparing it with different ablated versions. The paddy line test set is used for evaluation, and the results are presented in **Table 4**. From **Table 4**, we see that the addition of large convolutional kernels, at the tail of the encoder of *UNet* to capture a wider image context, improves the mIoU by 3.29%. Further, replacing the *UNet*-style fixed skip connections with the proposed multi-scale filter bank leads to an additional 3% improvement in mIoU.

**Table 4:** Ablation experiments to evaluate the effectiveness of the proposed extended skip connections.

Method	Baseline	Skip layer (fixed)	Large conv	Skip layer (multi-scale filters)	Paddy line IoU (%)
*UNet*	√	√			56.44
*UNet*_WC	√	√	√		59.73
**ESNet**	√		√	√	62.73

ESNet, enhanced skip network; IoU, intersection over union.

The motive behind using the multi-scale filters in the skip layers was to incorporate multi-scale features for reconstructing the output without having to rigidly set the convolutional kernel size. To verify this intuition, the multi-scale filter bank module is replaced with single-scale filters of size *k* × *k*. Different values of *k* ranging from 7 to 15 were evaluated, and the results are presented in **Table 5**.

**Table 5:** Performance comparison using different scales of filter in the skip layer.

*K*	7	9	11	13	15
**IoU (%)**	60.68	58.25	59.22	60.43	59.26

IoU, intersection over union.

From **Table 5**, we see that the network with *k* = 7 shows the best performance among the different single-scale filters evaluated. It can be observed that there is no straightforward relationship between the size of the kernel and network performance. From **Tables 4** and **5**, we see that the network with the proposed multi-scale filter bank outperforms all other networks with single-scale filters. The increased network capacity of the proposed filter bank may have led to increased accuracy. However, from **Table 5**, we see that increasing the network capacity by simply increasing the number of parameters does not necessarily improve the accuracy. The proposed structure allows the learning algorithm to choose either single-scale features or a combination of multi-scale features, whichever are efficient, and leads to better accuracy.

## Conclusion

In this study, we proposed a convolutional encoder–decoder network-based system to (a) extract the crop line and (b) differentiate between weeds and crops, in row-transplanted paddy fields. Different from the conventional methods of training DNNs, a novel method of training DNN using “semantic graphics” was proposed. Semantic graphics was introduced to annotate the target functional key-points, semantic regions, or other higher-level concepts which are otherwise challenging to annotate using existing bounding box-based or dense per-pixel-based approaches. An enhanced convolutional encoder–decoder network was then trained to directly learn the concept of crop line and discriminate between weeds and crop using semantic graphics.

Results demonstrating enhanced performance of the proposed method on the paddy line detection problem compared to other existing networks were presented. Experiments demonstrating enhanced performance of the proposed method on detecting paddy and wild millet compared to the more commonly used bounding box-based object detection approach were also presented.

The proposed crop line detection system can be easily extended to extract the rows of different types of crops. While the traditional handcrafted feature-based crop row extraction methods can fail to generalize well in real farm environments, the proposed crop line extraction system exhibits robust performance in real farm environments as demonstrated by the results. Though only wild millet detection is considered in this study, the proposed method can be extended easily to detect any other species of weeds. The crop lines extracted by the proposed method are accurate and can act as a reliable guiding line for an autonomous robot for inter-row weeding, whereas the detection of individual plants and weeds enables autonomous intra-row weeding. A combination of these two approaches for inter-row and intra-row weeding can be used to realize a comprehensive autonomous weeding system.

In the future, we plan to use the semantic graphics-based crop row detection method for vision-based control of an autonomous tractor for unmanned inter-row weeding in paddy and extend the system for intra-row weeding.

## Data Availability Statement

The datasets generated for this study are available on request to the corresponding author.

## Author Contributions

SA designed the study, collected the data, performed the experiments, analyzed the data, and wrote the paper. HY collected the data and performed the experiments. HK supervised and administered the overall project and reviewed and edited the writing.

## Funding

This work was supported in part by the Korea Research Fellowship Program through the National Research Foundation of Korea funded by the Ministry of Science and ICT (NRF-2015H1D3A1062316), and the Basic Science Research Program through the National Research Foundation of Korea (NRF) funded by the Ministry of Education (NRF-2019R1A6A1A09031717 and NRF-2019R1A2C1011297).

## Conflict of Interest

The authors declare that the research was conducted in the absence of any commercial or financial relationships that could be construed as a potential conflict of interest.
