# Supplementary material for: Learning Semantic Graphics Using Convolutional Encoder–Decoder Network for Autonomous Weeding in Paddy
Source: Front Plant Sci. 2019 Oct 31;10:1404. doi: 10.3389/fpls.2019.01404 (PMC6837080; doi:10.3389/fpls.2019.01404)
Supplement: Supplementary file 1 [file DataSheet_1.pdf]

Supplementary Material

1 Supplementary Figures

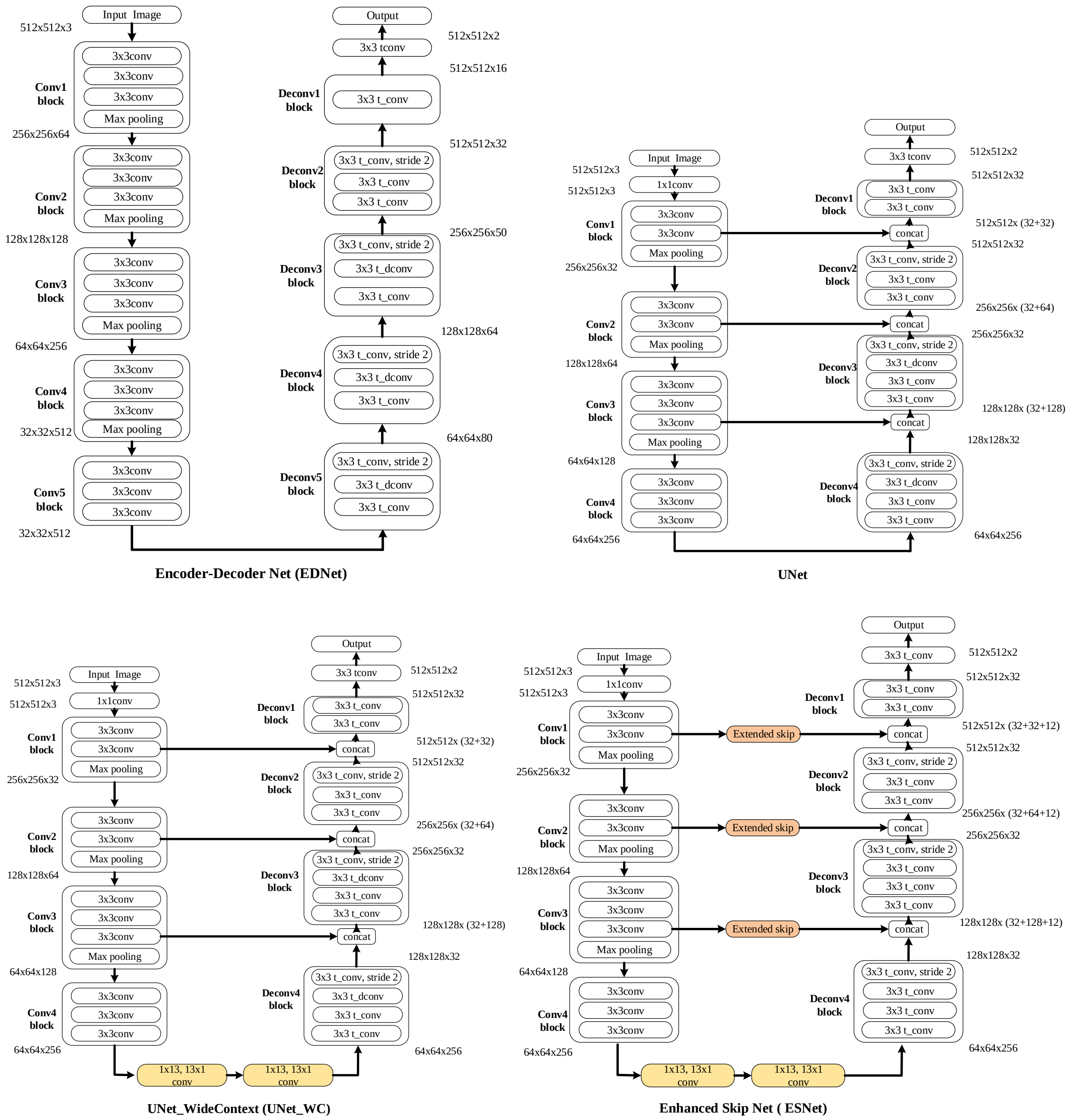

Supplementary Figure 1. Network architecture of (a) Encoder-decoder net (EDNet), (b) UNet, (c) Unet with wider context (UNet_WC), and (e) the proposed Extended Skip Network (ESNet).

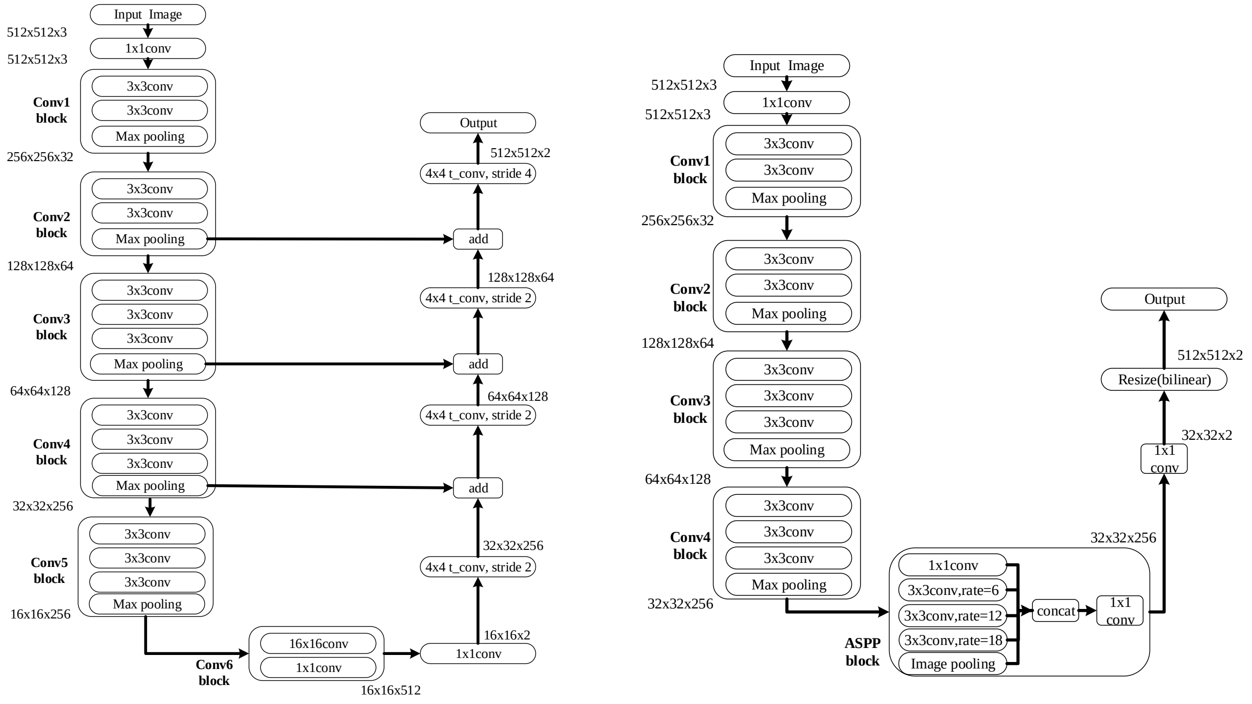

FCN8 **DeepLabV3**

Supplementary Figure 2. Network architecture of (a) FCN8 and (b) DeepLabV3 implemented for the experiments. (Convolution: conv, transposed convolution: t_conv, concatenation: concat)
